# TDP1 and TOP1 Modulation in Olaparib-Resistant Cancer Determines the Efficacy of Subsequent Chemotherapy

**DOI:** 10.3390/cancers12020334

**Published:** 2020-02-03

**Authors:** Jin Won Kim, Ahrum Min, Seock-Ah Im, Hyemin Jang, Yu Jin Kim, Hee-Jun Kim, Kyung-Hun Lee, Tae-Yong Kim, Keun Wook Lee, Do-Youn Oh, Jee-Hyun Kim, Yung-Jue Bang

**Affiliations:** 1 Department of Internal Medicine, Seoul National University College of Medicine, Seoul 03080, Korea; jwkim@snubh.org (J.W.K.); kyunghunlee@snu.ac.kr (K.-H.L.); ktyongmd@gmail.com (T.-Y.K.); imdoctor@snu.ac.kr (K.W.L.); ohdoyoun@snu.ac.kr (D.-Y.O.); jhkimmd@snu.ac.kr (J.-H.K.); bangyj@snu.ac.kr (Y.-J.B.); 2 Division of Hematology and Medical Oncology, Department of Internal Medicine, Seoul National University Bundang Hospital, Seongnam 13620, Korea; 3 Translational Medicine, Seoul National University College of Medicine, Seoul 03080, Korea; 4 Cancer Research Institute, Seoul National University, Seoul 03080, Korea; mar6716@snu.ac.kr (A.M.); applee63@nate.com (H.J.); dbwls0331@nate.com (Y.J.K.); 5 Biomedical Research Institute, Seoul National University Hospital, Seoul 03080, Korea; 6 Division of Hematology and Medical Oncology, Department of Internal Medicine, Seoul National University Hospital, Seoul 03080, Korea; 7 Department of Internal Medicine, Chung-Ang University College of Medicine, Seoul 06973, Korea; heejun@cau.ac.kr

**Keywords:** olaparib, carryover effect, TOP1 activity, TDP1, irinotecan

## Abstract

The aim of this study was to elucidate the carryover effect of olaparib to subsequent chemotherapy and its underlying mechanisms. We generated olaparib-resistant SNU-484, SNU-601, SNU-668, and KATO-III gastric cancer cell lines and confirmed their resistance by cell viability and colony forming assays. Notably, olaparib-resistant cell lines displayed cross-resistance to cisplatin except for KATO-III. Inversely, olaparib-resistant SNU-484, SNU-668, and KATO-III were more sensitive to irinotecan than their parental cells. However, sensitivity to paclitaxel remained unaltered. There were compensatory changes in the ATM/ATR axis and p-Chk1/2 protein expression. ERCC1 was also induced in olaparib-resistant SNU-484, SNU-601, and SNU-668, which showed cross-resistance to cisplatin. Olaparib-resistant cells showed tyrosyl-DNA phosphodiesterase 1 (TDP1) downregulation with higher topoisomerase 1 (TOP1) activity, which is a target of irinotecan. These changes of TOP1 and TDP1 in olaparib-resistant cells was confirmed as the underlying mechanism for increased irinotecan sensitivity through manipulated gene expression of TOP1 and TDP1 by specific plasmid transfection and siRNA. The patient-derived xenograft model established from the patient who acquired resistance to olaparib with BRCA2 mutation showed increased sensitivity in irinotecan. In conclusion, the carryover effects of olaparib to improve antitumor effect of subsequent irinotecan were demonstrated. These effects should be considered when determining the subsequent therapy with olaparib.

## 1. Introduction

Olaparib is an oral poly(ADP-ribose) polymerase (PARP) inhibitor that traps inactivated PARP onto single-strand DNA breaks (SSBs), leading to double-strand DNA breaks that induce cell death [1,2]. Olaparib showed antitumor effect alone or in combination with chemotherapy in tumors with deficiencies at the repair of double-strand breaks caused by BRCA1/2 mutations, ATM dysfunction, and RAD51C deficiency [3,4,5,6]. Recently, in germline BRCA-mutated advanced ovarian cancer patients who have been treated with three or more prior lines of chemotherapy, olaparib showed clinical efficacy and has been approved by U.S. Food and Drug Administration [4]. The Cancer Genome Atlas reported that 7 of 287 gastric patients (2.4%) showed genetic alterations in RAD51C gene, and it has been well established that loss of RAD51C is associated with increased cancer risk [7,8,9]. In our previous study, we found that RAD51C depletion led to hypersensitivity to olaparib in gastric cancer cells [6]. Therefore, PARP inhibition can be an attractive strategy for gastric cancer treatment.

In a randomized phase II trial of second-line metastatic gastric cancer, the addition of olaparib to paclitaxel showed much longer overall survival (OS) than paclitaxel plus placebo, although the progression-free survival (PFS) was marginally different between the two groups [10]. This finding was also identified in a randomized phase 3 trial of using olaparib in second-line gastric cancer [11]. It means that post-progression survival was longer in olaparib arm; however, the reason for this could not be fully explained. There is no preclinical evidence to explain how olaparib affects post-progression survival. In a previous study, our group showed the prolonged antitumor effect of olaparib even after stopping olaparib treatment in an in vivo xenograft model [6]. Based on this finding, carryover effect of olaparib, which has an effect on subsequent chemotherapy, was suggested as one of the potential explanations for this, particularly, in irinotecan, which is usually applied as a third line of treatment.

PARP-1 is activated by irinotecan-induced DNA breaks. PARP inhibitors share their predictive characters with platinum and enhance the cytotoxicity of topoisomerase 1 (TOP1) inhibitors [12]. Restoration of homologous recombination through a loss of 53BP1 or BRCA re-expression is an important mechanism for PARP inhibitor resistance in BRCA1-deficient mammary tumors [13]. A previous study has demonstrated that ATM regulates TOP2 expression. ATM loss results in increased TOP2 levels and enhances sensitivity to TOP2 inhibition [14]. Therefore, it could be possible to compensate for an impaired DNA repair pathway from olaparib by another repair-pathway related to SSB, including the TOP1 and tyrosyl-DNA phosphodiesterase 1 (TDP1) pathways, in resistant cell lines that have been exposed to olaparib for long periods. On the other hand, it was reported that TDP1 and TOP1 are predictive biomarkers for irinotecan and new therapeutic targets in the era of precision medicine [14,15].

On these backgrounds, we explored the carryover effect of olaparib, which influences the efficacy of subsequent chemotherapy after olaparib treatment. Additionally, we attempted to evaluate the mechanisms underlying this effect.

## 2. Results

### 2.1. Altered Sensitivity of Chemotherapeutic Agents in Olaparib-Resistant Cells

**Figure 1:**
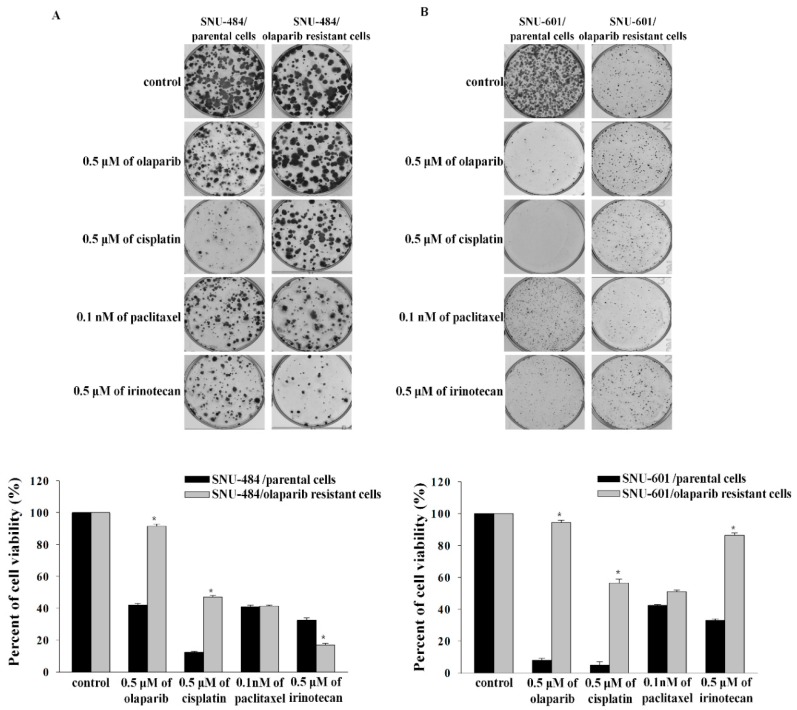

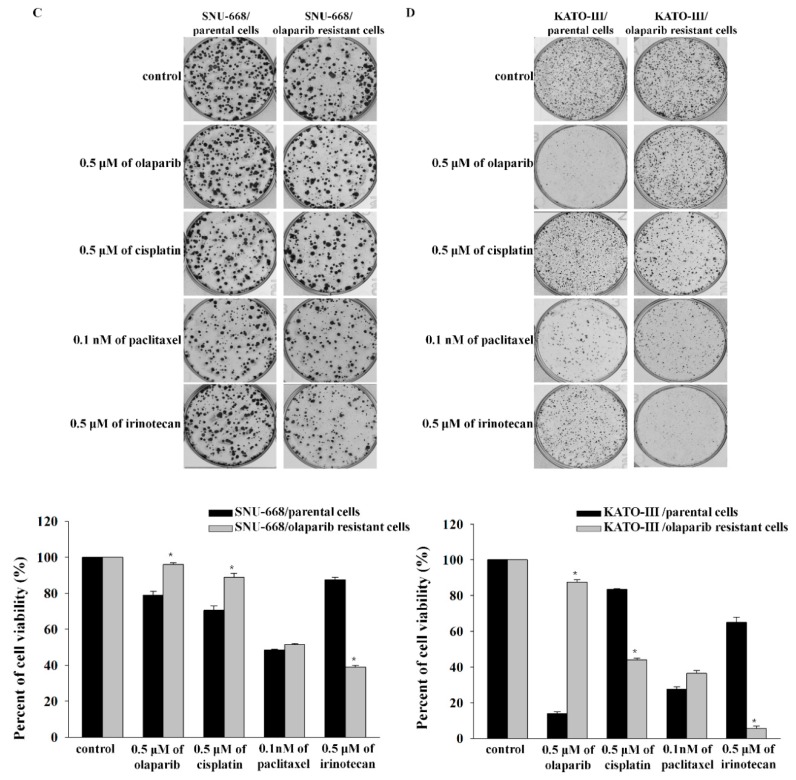
The sensitivity of chemotherapeutic agents was altered in olaparib-resistant cells. Cell viability was calculated through counting cell colonies by using GELCOUNT in SNU-484 (**A**), SNU-601 (**B**), SNU-668 (**C**), and KATO-III (**D**) cells. * indicates *p* < 0.001.

We established olaparib-resistant cell lines by long-term treatment with olaparib from SNU-484, SNU-601, SNU-668, and KATO-III. The resistance was confirmed by using cell growth inhibition and colony forming assays (Table 1 and Figure 1). Except for KATO-III, all olaparib-resistant cell lines became resistant to cisplatin. Inversely, the olaparib-resistant cell lines became more sensitive to irinotecan, except for SNU-601. Apoptosis also increased after irinotecan treatment in olaparib-resistant cell lines (Figure S1). Sensitivity toward paclitaxel was not altered after the acquisition of resistance to olaparib.

### 2.2. Changes of Proteins Related to the DNA-Damage Response in Olaparib-Resistant Cell Lines

**Figure 2:**
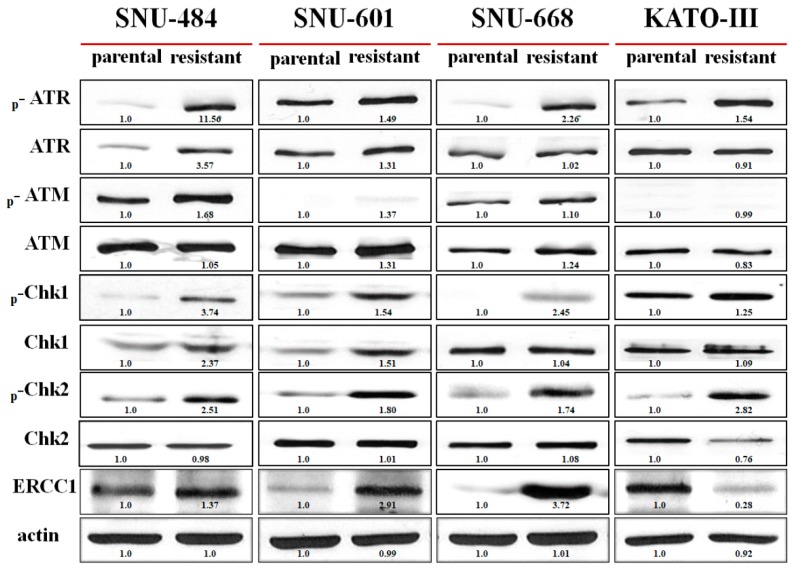
The expressions of DNA-damage response proteins were altered after acquisition of olaparib resistance. Western blot analysis for p-ATR, ATR, p-ATM, ATM, p-Chk1, Chk1, p-Chk2, Chk2, and ERCC1 was conducted to evaluate the altered expression after acquisition of resistance to olaparib. There were compensatory changes in the DNA-damage response proteins.

To evaluate the mechanisms underlying the altered sensitivity toward cisplatin, changes of the DNA-damage response proteins were evaluated. p-ATR was induced in all olaparib-resistant cell lines (Figure 2). p-ATM was slightly upregulated in olaparib-resistant SNU-484 and olaparib-resistant SNU-668. p-Chk1 and p-Chk2 were elevated in olaparib-resistant cell lines compared with the parental cells. Furthermore, ERCC1 was also upregulated in olaparib-resistant SNU-484, SNU-601, and SNU-668 cells, which were resistant to cisplatin. This ERCC1 induction was not detected in olaparib-resistant KATO-III cells, which were not resistant to cisplatin.

### 2.3. Morphological Changes in the Olaparib-Resistant Cell Lines

**Figure 3:**
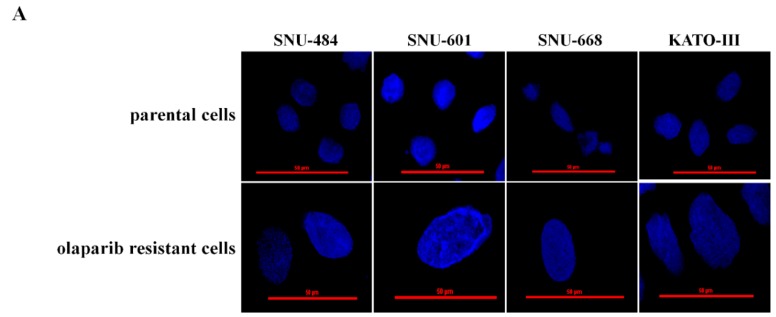

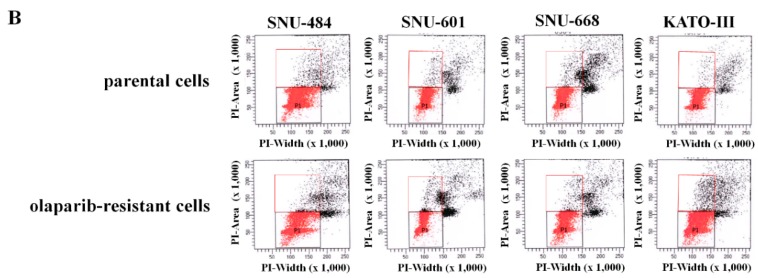
The nuclei were larger in olaparib-resistant cells than in parental cells. The degree of DNA condensation was examined by 4′,6-diamidino-2-phenylindole (DAPI) staining and confocal laser microscopy. Representative images are presented (80× original magnification). Scale bars represent 50 μm. In olaparib-resistant cells, increased nucleus indicates a relaxation of DNA condensation (**A**). The proportion of cells with a DNA content of more than 4n was calculated by flow cytometry with PI staining. The increased nuclear size of olaparib-resistant cells was not related to aneuploidy (**B**).

With the acquisition of resistance to olaparib, the nuclear size of the resistant cells increased, as assessed by confocal imaging (Figure 3A). Increased abnormal DNA contents indicating aneuploidy was not observed in cells with increased nuclear size (Figure 3B). In the olaparib-resistant cells, increased nuclear size indicated the relaxation of DNA condensation [16,17].

### 2.4. Increased TOP1 Activity and Decreased TDP1 Expression in Olaparib-Resistant Cell Lines

**Figure 4:**
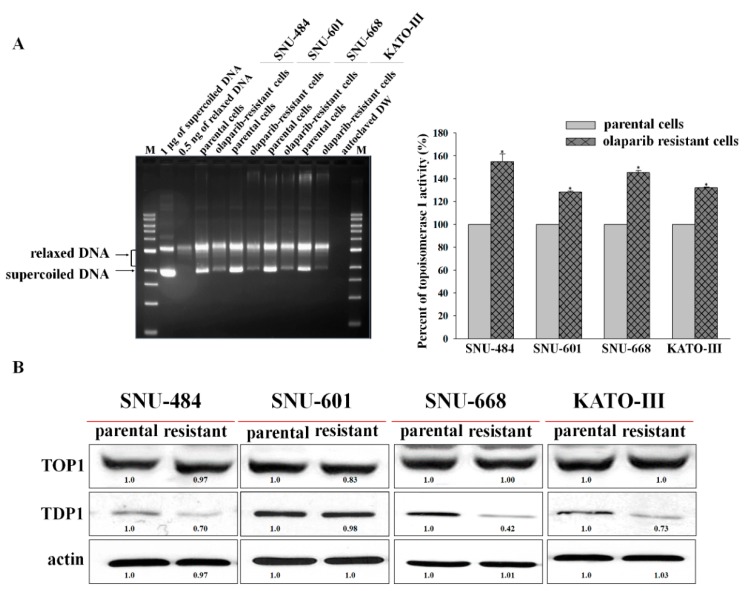
Topoisomerase 1 (TOP1) activity was increased and tyrosyl-DNA phosphodiesterase 1 (TDP1) expression was downregulated in olaparib-resistant cells. TOP1 activity was increased in all olaparib-resistant cells compared with parental cells (**A**), although the protein expression of TOP1 was not altered (**B**). TDP1 protein expression was downregulated in olaparib-resistant SNU-484, SNU-668, and KATO-III cells compared with their parental cells (**B**). * indicates *p* < 0.001.

To evaluate the underlying mechanism of increased irinotecan sensitivity in the olaparib-resistant cell lines, protein expression, and activity of TOP1 and protein expression of TDP1, which are the target and predictive markers of irinotecan, were measured. Olaparib-resistant cells showed higher TOP1 activity than their parental cells, although protein expression of TOP1 was not altered (Figure 4A,B). In addition, TDP1 expression was decreased in olaparib-resistant cell lines except in olaparib-resistant SNU-601, which did not exhibit sensitivity to irinotecan and had a mutation in TDP1 (Table 2).

### 2.5. Changes of Irinotecan Sensitivity According to Manipulated TOP1 and TDP1 Expression

**Figure 5:**
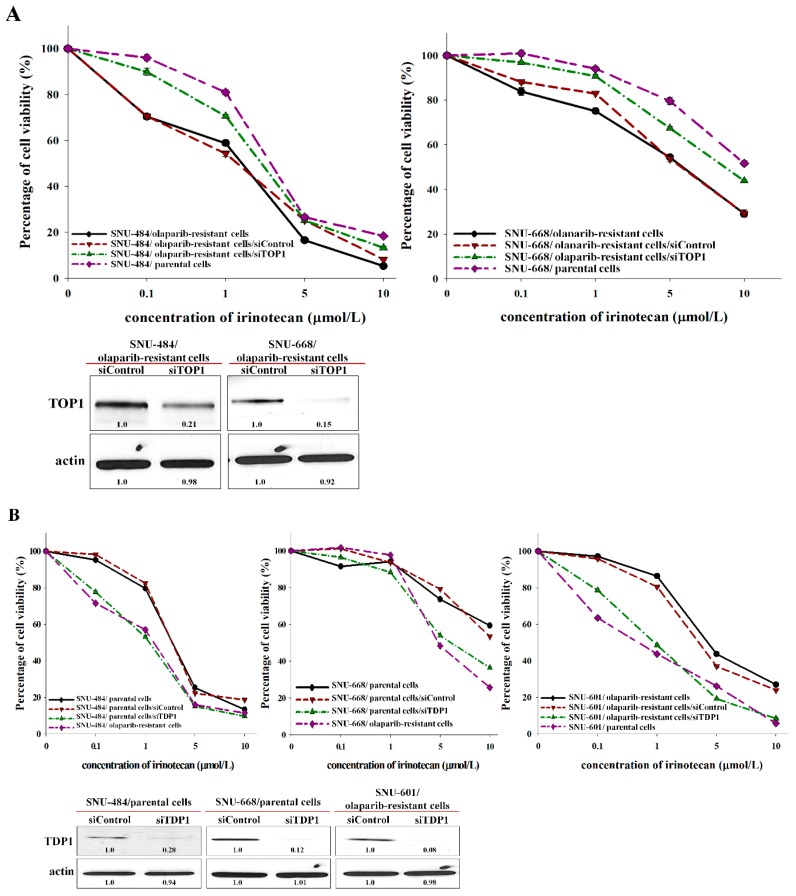

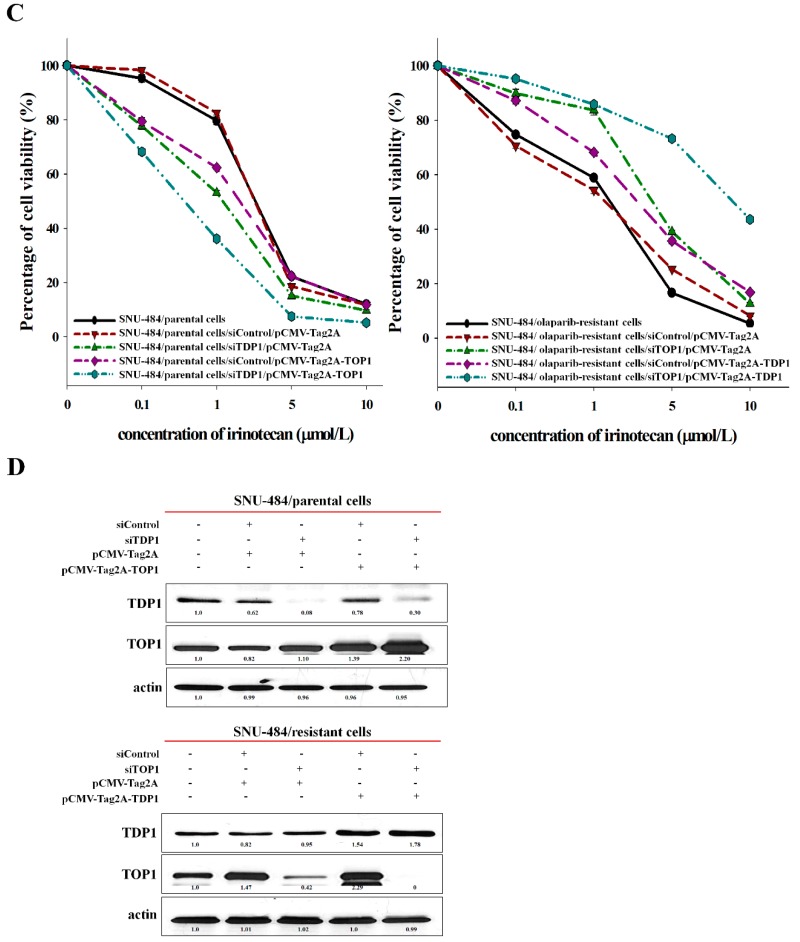
The sensitivities to irinotecan with modulating TOP1 and TDP1 expressions were evaluated by MTT assay. TOP1-siRNA mix, TDP1-siRNA mix, and non-targeting siRNA (160nM each) were transfected into olaparib-resistant cells or parental cells, and then treated with irinotecan for 5 d. Cell viability percentages were then measured by MTT assay, and TOP1 or TDP1 silencing was determined by western blotting (**A**,**B**). pCMV-tag2A-TOP1 or pCMV-tag2A-TDP1 and/or siTOP1 or siTDP1 were transfected into parental or olaparib-resistant cells. The sensitivity to irinotecan was calculated by MTT assay and the efficacy of transfection was confirmed by western blotting (**C**,**D**).

**Figure 6:**
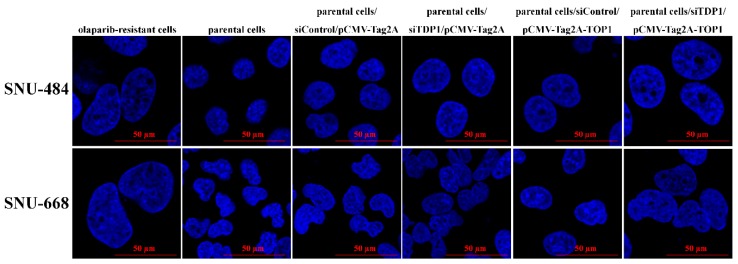
Parental cells were transfected with siRNA targeting TDP1 and/or pCMV-tag2A-TOP1 for 5 d. The size of nuclei was measured by DAPI staining and confocal laser microscopy. Representative images are presented (80× original magnification). Scale bars represent 50 μm.

To determine if the manipulation of TOP1 and TDP1 could change the sensitivity of irinotecan, the sensitivity of irinotecan was evaluated according to manipulated expressions of TOP1 and TDP1 by using specific plasmid transfection and siRNA. In olaparib-resistant cells of SNU-484 and SNU-668 with increased activity of TOP1, irinotecan sensitivity was reduced when TOP1 expression was downregulated by siRNA (Figure 5A). In addition, in parental cells of SNU-484 and SNU-668, decreased TDP1 expression by siRNA induced the increased irinotecan sensitivity as same as olaparib-resistant cells. Olaparib-resistant SNU-601, in which TDP1 expression was not decreased, also showed an increased irinotecan sensitivity when TDP1 expression was knocked down (Figure 5B). Inversely, TOP1, or TDP1, expression was overexpressed by plasmid transfection in parental or olaparib-resistant cells of SNU-484 (Figure 5C). Although overexpressed TOP1 had slightly increased irinotecan sensitivity, simultaneous upregulation of TOP1 with downregulation of TDP1 in parental SNU-484 showed the significantly increased sensitivity to irinotecan compared with parental cells (Figure 5C). Furthermore, transiently overexpressed TDP1 had attenuated irinotecan sensitivity in olaparib-resistant SNU-484 cells, but olaparib-resistant SNU-484 with TOP1 downregulation and TDP1 upregulation was confirmed to attenuate the sensitivity to irinotecan dramatically (Figure 5C). The successful expression modulation by transfection was validated by western blot analysis (Figure 5C). Therefore, olaparib-resistant cell lines could be highly sensitive to subsequent irinotecan through TDP1 downregulation at the same time as the increased TOP1 activity. Furthermore, downregulation of TDP1 with TOP1 upregulation increased the nuclear size in parental cells, similar to olaparib-resistant cells (Figure 6). Thus, morphological changes of olaparib-resistant cells could be attributed to the relaxation of DNA condensation modulated by TOP1 hyperactivity and depletion of TDP1.

### 2.6. Potent Anti-Tumor Activity of Irinotecan in Olaparib-Resistant Patient-Derived Breast Cancer Xenograft Model

**Figure 7:**
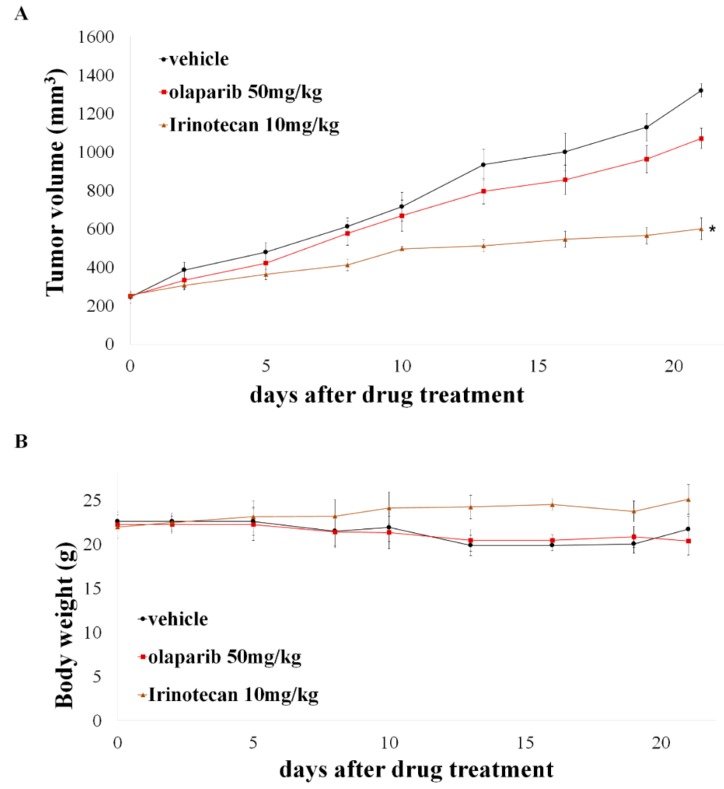
Irinotecan impeded tumor growth in a BRCA2 mutant PDX model with acquisition of olaparib resistance. (**A**) PDX model were treated with 50 mg/kg olaparib or 10 mg/kg irinotecan for 21 days (n = 4/group). The mean of tumor volumes was presented in a graph with the standard deviation (SD). * indicates *p* < 0.001 versus vehicle and olaparib group. (**B**) Changes in body weight were measured every three days for 21 days.

To validate the increased antitumor effect of irinotecan in olaparib resistant model, we tested the antitumor activity of irinotecan in patient-derived xenograft (PDX) models established from a breast cancer patient with BRCA2 580del4 mutation who acquired resistance to olaparib. In concordance with clinical response, IMX 181 model showed no response to olaparib, but statistically significant delay of tumor growth on irinotecan treatment (Figure 7A). Both treatments were well tolerated without any sign of toxicity (Figure 7B).

## 3. Discussion

This study confirmed the carryover effect of olaparib-treatment to subsequent chemotherapy, particularly in irinotecan. Through manipulating gene expressions, increased sensitivity to irinotecan in olaparib-resistant cells was confirmed to be attributed to TOP1 upregulation and TDP1 downregulation, which was shown in olaparib-resistant cells. These results could explain the higher OS improvement compared with PFS prolongation in the randomized clinical study of second-line gastric cancer, evaluating the efficacy of olaparib combined with paclitaxel.

After irinotecan treatment, SSBs are repaired by a complex consisting of TDP1, which functions in the base excision repair pathway [18]. PARP inhibitors, which inhibit base excision repair, sensitize cells to TOP1 inhibitors [19]. Therefore, irinotecan and olaparib represent a potent combination. However, concurrent treatment with both PARP inhibitors and irinotecan is too toxic for clinical development, although a preclinical study demonstrated synergistic effects [20,21,22,23]. Therefore, sequential treatment might represent a promising alternative approach. Our results suggest that the application of irinotecan after olaparib treatment may be a feasible treatment option owing to the carryover effect of olaparib.

DNA-damage response proteins function in various complex and overlapping pathways. In the present study, long-term olaparib treatment induced a compensatory alteration of the ATR/ATM axis, Chk, and ERCC1 expression. The development of olaparib resistance can be ascribed to this compensatory alteration, which also results in cisplatin resistance. Furthermore, olaparib and platinum share a common mechanism of action in the DNA repair pathway and similar predictive characters, such as the presence of BRCA mutation and RAD51 deficiency [12,24,25,26]. For a specific example, SNU-601 was highly sensitive to olaparib due to RAD51C-deficiency, which was also identified in several cancer types [6,25,26]. Parental SNU-601 was also sensitive to cisplatin, and olaparib-resistant SUN-601 showed resistance to cisplatin likewise. Therefore, this suggests that treatment with platinum should be avoided after olaparib failure, although clinical studies have so far not shown a decreased response to platinum after the resistance of PARP inhibitor in ovarian cancer [27]. In addition, this carryover effect might be specific to tumor cells based on synthetic lethality as a cytotoxic mechanism of PARP inhibitor. Clinically, there are no studies to report that this carryover effect could exacerbate the toxicities of subsequent chemotherapy.

TOP1 is an important cellular enzyme that allows for DNA relaxation. TOP1 cleaves DNA to create a DNA single-strand break to which it remains covalently bound to, thus allowing for rotation and relaxation of DNA. Once rotated, bound TOP1 ligates the nicked DNA and is released. In olaparib-resistant cell lines, the size of the nucleus was larger than that in the parental cell lines. This finding gave an indirect clue that TOP1 activity was increased in olaparib-resistant cells [16,17]. TOP1 activity is a predictive marker of irinotecan [28,29]. In the present study, increased TOP1 activity in olaparib-resistant cells was confirmed by using TOP1 activity assay. According to changes of TOP1 expression by transfection of siRNA, the sensitivity of irinotecan was changed, and the size of the nucleus was altered corresponding to olaparib-resistant cells. These results, therefore, provide direct evidence for the alteration of irinotecan sensitivity in olaparib-resistant cells.

TOP1 activity was increased in all olaparib-resistant cell lines, although TOP1 protein expression was not altered. TDP1 expression was downregulated in olaparib-resistant SNU-484, SNU-668, and KATO-III cells, which were more sensitive to irinotecan after the acquisition of olaparib resistance. Exceptionally, olaparib-resistant SNU-601 did not show altered expression of TDP1 and sensitivity to irinotecan. SNU-601 had TDP1 mutation (A520D). It has been reported that inactive mutant TDP1 (H263A) did not reduce DNA-damage by camptothecin, although the function of this TDP1 mutation (A520D) was unknown [30]. In colon cancer, TDP1 depletion increased the sensitivity to irinotecan in a TOP1-dependent manner [31]. In our study, in parental cells, TOP1 upregulation and TDP1 downregulation resulted in the highest sensitivity to irinotecan compared with either change alone. The mechanism underlying the carryover effect of olaparib for irinotecan could be confirmed, as these changes in olaparib-resistant cells, which included an increased TDP1 downregulation along with increased TOP1 activity.

The PDX model established from the patient who acquired resistance even harboring BRCA2 mutation exhibited sensitivity to irinotecan. These results can help to explain how olaparib showed an increase in overall survival, without increasing PFS in phase 2 trial conducted in gastric cancer, by its carryover effect on irinotecan, which is used as a third-line treatment [10].

In summary, this study demonstrated that there were carryover effects after acquisition of olaparib resistance. In olaparib-resistant cells, cisplatin resistance might occur because of compensatory alterations in the ATR/ATM axis, and Chk and ERCC1 expression. Importantly, irinotecan sensitivity was enhanced through TDP1 downregulation, concomitant with increased TOP1 activity in olaparib-resistant cells. Sensitivity to paclitaxel remained unaltered after acquisition of resistance to olaparib. Based on these results, carryover effect of olaparib to subsequent therapy should be significantly considered during the clinical use of olaparib.

## 4. Materials and Methods

### 4.1. Reagents

The PARP inhibitor, olaparib, was kindly provided by AstraZeneca, Ltd. (Macclesfield, Cheshire, UK). Cisplatin and paclitaxel were obtained from Choongwoe Co., Ltd., and Samyang Genex Co., Ltd. (Seoul, Korea). Irinotecan (cas no. 100286-90-6) was purchased from Sigma Aldrich (St. Louis, MO, USA).

### 4.2. Cell Lines and Cell Culture

Human gastric cancer cells (SNU-484, SNU-601, SNU-668, and KATO-III) were purchased from the Korean Cell Line Bank (Seoul, Korea). The identities of cell lines were validated by DNA fingerprinting analysis [32]. The cells were cultured at 37 °C in an atmosphere containing 5% CO_2_ in Roswell Park Memorial Institute (RPMI) 1640 supplemented with 10% fetal bovine serum (FBS) and 10 μg/mL gentamicin.

### 4.3. Generation of Olaparib-Resistant Cells

Olaparib-resistant SNU-484, SNU-601, SNU-668, and KATO-III cells were established by continuous exposure to olaparib, starting with 1 μmol/L and incrementally increasing the concentration to 5 μmol/L over 7 months. Resistant cells were expanded in RPMI-1640 medium containing 10% FBS and 1 μmol/L olaparib.

### 4.4. Cell Growth Inhibition Assay

Cells (2–3 × 10^3^ in 100 μL/well) were seeded in 96-well plates and incubated overnight at 37 °C in 5% CO_2_, and then exposed to each drug at the indicated concentration for 5 days. After drug treatment, 50 μL of 3-(4,5-dimethylthiazol-2-yl)-2,5-diphenyltetrazolim bromide solution (Sigma Aldrich) was added to each well, and the plates were incubated for 4 h at 37 °C before the media was removed. After dissolving the formazan crystals with 150 μL of dimethyl sulfoxide, the absorbance of each well was measured at 540 nm with a VersaMax™ microplate reader (Molecular Devices, Sunnyvale, CA, USA). Half-maximal inhibitory concentration (IC_50_) values were analyzed using SigmaPlot software (Statistical Package for the Social Sciences, Inc., Chicago, IL, USA).

### 4.5. Colony-Formation Assay

To compare the drug response between parental cells and resistant cells, the cells were seeded into six-well plates and incubated with the indicated concentration of each drug for 14 days. The cell colonies were washed in phosphate-buffered saline and stained with 0.1% Coomassie Blue solution (Sigma Aldrich) for 1 h at room temperature. The excess staining solution was then removed, and the plates were washed in PBS and air-dried. The cell colonies were counted using GELCOUNT (Oxford Optronix Ltd., Abingdon, UK), and cell viability was calculated by using SigmaPlot.

### 4.6. Western Blot Analysis

Whole cell proteins were extracted by using RIPA buffer, and equal amounts of protein were separated on 5%–15% SDS-PAGE gels, as described previously [33]. Primary antibodies against phosphorylated (p)-ATM, ATM, p-ATR, ATR, p-Chk1, Chk1, p-Chk2, Chk2, and ERCC1 were acquired from Cell Signaling Technology (Beverley, MA, USA). Antibodies against TOP1 and TDP1 were purchased from Abcam (Cambridge, UK). Actin antibody (Sigma Aldrich) was used as a control. The band intensity was calculated by Image J software.

### 4.7. Immunofluorescence Assay

The degree of DNA condensation was examined by 4′,6-diamidino-2-phenylindole (DAPI) staining and confocal laser microscopy. Cells were plated on 0.01% poly-l-lysine (Sigma Aldrich)-coated coverslips and incubated overnight. Then, the cells were stained with DAPI (300 nM; Invitrogen, Carlsbad, CA, USA) for 1 min. The coverslips were rinsed three times for 10 min in PBS and mounted on slides using Faramount aqueous mounting medium (DAKO, Denmark). Immunofluorescence was visualized using a Zeiss LSM 510 laser-scanning microscope.

### 4.8. Nuclear Extraction

Cells were incubated in hypotonic buffer A (10 mM HEPES (pH 7.5), 1.5 mM MgCl_2_, 10 mM KCl, 0.2 mM EDTA, 0.5 mM DTT, 2 mM phenylmethylsulfonyl fluoride (PMSF), 1 mg/mL pepstatin A, 0.2 mM leupeptin, 10 μg/mL aprotinin, 1 mM sodium vanadate, 1 mM nitrophenylphosphate, and 5 mM benzamidine) for 10 min at 4 °C. The cells were incubated with 0.1% NP-40 for 10 min at 4 °C, and then centrifuged at 2000 rpm for 5 min at 4 °C. The supernatant was removed, and the pellets were resuspended in 1 mL of cold TEM solution (10 mM Tris-HCl (pH 7.5), 1 mM EDTA, and 4 mM MgCl_2_) with 0.5 mM PMSF and centrifuged at 2000 rpm at 4 °C. The nuclear pellets were resuspended in a small volume of TEM, and mixed and incubated with an equal volume of 1 M NaCl for 30 min at 4 °C. Then, the supernatant was kept as the nuclear extract, following centrifugation at 13000 rpm for 15 min at 4 °C.

### 4.9. TOP1 Activity Assay

The TOP1 activities of the nuclear extracts from each cell line were assessed by measuring the relaxation of the supercoiled pHOT1 plasmid (TopoGEN, Inc., Buena Vista, CO, USA). Supercoiled pHOT1 plasmid (250 ng/μL) was incubated with 1 μL of nuclear extract in 10 mM Tris-HCl (pH 7.9), 150 mM NaCl, and 1 mM EDTA for 60 min at 37 °C in a final volume of 20 μL [34,35]. The reaction was terminated by the addition of 4 μL of stop buffer (1% Sarkosyl, 0.025% bromophenol blue, and 5% glycerol), and the samples were loaded onto 1% agarose gels. TOP1 activities were calculated by measuring the degree of disappearance of the supercoiled DNA and are presented in a bar graph.

### 4.10. Plasmid and siRNA Transfection

pME18S-FL3-TOP1 and pOTB-TDP1 plasmids were purchased from the Korean human gene bank (Daejoen, Korea) and both TOP1 and TDP1 were subcloned into pCMV-Tag2A vector. SNU-484 and SNU-668 parental cells were transfected with 4 µg of pCMV-Tag2A-TOP1 plasmid and/or 160 nM of TDP1-specific siRNA, while SNU-484 and SNU-668 olaparib resistant cells were transfected with 4µg of pCMV-Tag2A-TDP1 plasmid and/or 160 nM of TOP1-specific siRNA using Lipofectamin 2000 (Invitrogen, Carlsbad, CA, USA). TOP1- and TDP1-specific siRNAs were synthesized from Genolution (Seoul, Korea), and the sequences of the TOP1-specific siRNAs were 5′-GGGAAGGACTCCATCAGATACTATA-3′, 5′-AAGTGGAAATGGTGGGAAGAA-3′, and 5′-CGAAGAAGGTAGTAGAGTC-3′, and the sequences of the TDP-specific siRNAs were 5′-GACCATATCTAGTAGTGAT-3′, 5′-GGAGTTAAGCCAAAGTATA-3′, and 5′-CTAGACAGTTTCAAAGTG-3′. The sequence of the non-specific siRNA was 5′-AATTCTCCGAACGTGTCACG-3′.

### 4.11. Library Preparation and Sequencing

Library preparations, clustering, and whole genome sequencing were conducted by Macrogen (Seoul, Korea). Libraries were prepared according to the TruSeq nano DNA library prep manual (Illumina; San Diego, CA, USA). Genomic DNA (100 ng) was sheared using an LE220 Focused-ultrasonicator (Covaris; Woburn, MA, USA) with a duty factor of 15%, peak incident power of 450 W, 200 cycles per burst for 50 s. Sheared DNA fragments of around 350 bp were obtained according to the manufacturer’s instructions. DNA libraries were enriched after ligating the indexing adapters to the ends of the DNA fragments. The quality of the libraries was evaluated using TapeStation 2200 (Agilent; Santa Clara, CA, USA), quantified by using PicoGreen^®^ dsDNA quantitation assay (Thermo Fisher Scientific; Waltham, MA, USA), and measured on a Victor3 plate reader (PerkinElmer; Waltham, MA, USA). A unique “bridged” amplification reaction was utilized for sequencing. A flow cell containing the libraries was prepared and then loaded on to the HiSeq X-10 sequencer (Illumina) for automated cycles of extension and imaging.

### 4.12. Patient-Derived Xenograft (PDX) Study

Animal experimentation was performed in accordance with the guidelines approved by the Institutional Animal Care and Use Committee (IACUC) and IRB (20180416-1402-054-555). Fresh human breast cancer tissue was obtained through a gun biopsy from hormone-receptor-positive, HER2-negative IDC breast cancer patient with *BRCA2* 580del4 mutation, who acquired resistance to olaparib. The obtained tissue was cut ~2-mm pieces within 1 h while covered in gauze dipped in saline and transplanted into six-week-old, severe combined immunodeficient (NOG) female mice (F0). Fresh human breast cancer tissue was cut into ~2-mm pieces and transplanted into 6-week-old, severe combined immunodeficient (NOG) female mice (F0). When the tumor volumes reached to 1.5 cm in diameter, it was dissected and re-implanted into another set of mice (F1). When the tumor volume of F1 reached 150 mm^3^, the mice were randomly divided into three groups (4 mice per each group) and olaparib (50 mg/kg, p.o, once daily), irinotecan (10 mg/kg, i.p, twice a week), or vehicle (0.5% hydroxypropyl methylcellulose, p.o, once daily) were administered. Tumor volume was measured three times a week by caliper and calculated using ((width)^2^ × (height))/2. All mice were sacrificed with CO_2_ on 21 days after the treatment.

### 4.13. Statistical Analysis

All experiments were conducted independently at least three times, and statistical analyses were performed using SigmaPlot version 9.0 (Statistical Package for the Social Sciences, Chicago, IL, USA). Two-sided Student’s t-test was used when appropriate. The results are expressed as the mean ± standard error (SE). A *p*-value < 0.01 was considered to represent statistical significance.

### 4.14. Ethics Declarations

The PDX study was approved by the Institutional Animal Care and Use Committee (IACUC) and IRB (20180416-1402-054-555).

## 5. Conclusions

A carryover effect of olaparib-treatment, sensitizing to subsequent treatment, was suggested in a clinical trial. To explore the possibility and the mechanism of this effect, olaparib-resistant gastric cancer cells were tested with several chemotherapeutic agents, and underlying mechanisms were explored. Olaparib-resistant gastric cancer cells show the compensatory alterations in DNA-damage response pathways, and exhibit cross-resistance to cisplatin; however, these cells are highly sensitive to subsequent treatment with irinotecan through tyrosyl-DNA phosphodiesterase 1 (TDP1) downregulation with increased topoisomerase 1 (TOP1) activity. These findings have substantial implications on subsequent therapies for patients with olaparib-resistant cancers.

## Figures and Tables

**Table 1:** Drug sensitivity in parental and olaparib-resistant cells, as assessed by using cell growth-inhibition assay.

Cell Lines *		Olaparib IC_50_ (μmol/L, mean ± SD)	Cisplatin IC_50_ (μmol/L, mean ± SD)	Paclitaxel IC_50_ (μmol/L, mean ± SD)	Irinotecan IC_50_ (μmol/L, mean ± SD)
SNU-484	Parental cells	4.16 ± 0.05	0.8 ± 0.01	2.7 ± 0.3	3.96 ± 0.2
	Olaparib-resistant cells	>10	2.02 ± 0.03	2.5 ± 0.08	1.47 ± 0.2
SNU-601	Parental cells	0.73 ± 0.006	0.75 ± 0.005	4.63 ± 0.08	1.053 ± 0.03
	Olaparib-resistant cells	7.3 ± 0.4	3.92 ± 0.05	4.92 ± 0.05	1.82 ± 0.007
SNU-668	Parental cells	11.07	5.88 ± 0.2	5.35 ± 0.1	>10
	Olaparib-resistant cells	>20	>10	5.66 ± 0.1	5.95 ± 0.1
KATO-III	Parental cells	3.56 ± 0.4	>10	5.67 ± 0.1	>10
	Olaparib-resistant cells	>10	4.13 ± 0.4	5.82 ± 0.1	5.5 ± 0.4

* MTT for 5 days.

**Table 2:** Mutation status for *TDP1* and *TOP1* in parental and olaparib-resistant cells.

Cell Line	*TDP1*	*TOP1*
SNU-484/parental cells	wild type	wild type
SNU-484/olaparib-resistant cells	wild type	wild type
SNU-601/parental cells	wild type	wild type
SNU-601/olaparib-resistant cells	A520D	wild type
SNU-668/parental cells	wild type	wild type
SNU-668/olaparib-resistant cells	wild type	wild type
KATO-III/parental cells	wild type	wild type
KATO-III/olaparib-resistant cells	wild type	wild type

TDP1, tyrosyl-DNA phosphodiesterase 1; TOP1, topoisomerase 1.

## Supplementary Materials

The following are available online at https://www.mdpi.com/2072-6694/12/2/334/s1, Figure S1. The populations of apoptotic cells following irinotecan or olaparib treatment; Figure S2. Protein expression levels of parental and olaparib-resistant cells; Table S1. Sequences of the siRNAs.
