# Supplementary material for: TDP1 and TOP1 Modulation in Olaparib-Resistant Cancer Determines the Efficacy of Subsequent Chemotherapy
Source: Cancers (Basel). 2020 Feb 3;12(2):334. doi: 10.3390/cancers12020334 (PMC7072281; doi:10.3390/cancers12020334)
Supplement: Supplementary file 1 [file cancers-12-00334-s001.pdf]

cancers

MDPI

TDP1 and TOP1 Modulation in Olaparib-Resistant Cancer Determines the Efficacy of Subsequent Chemotherapy

Jin Won Kim, Ahrum Min, Seock-Ah Im, Hyemin Jang, Yu Jin Kim, Hee-Jun Kim, Kyung-Hun Lee, Tae-Yong Kim, Keun Wook Lee, Do-Youn Oh, Jee-Hyun Kim and Yung-Jue Bang

Supplementary Figure S1

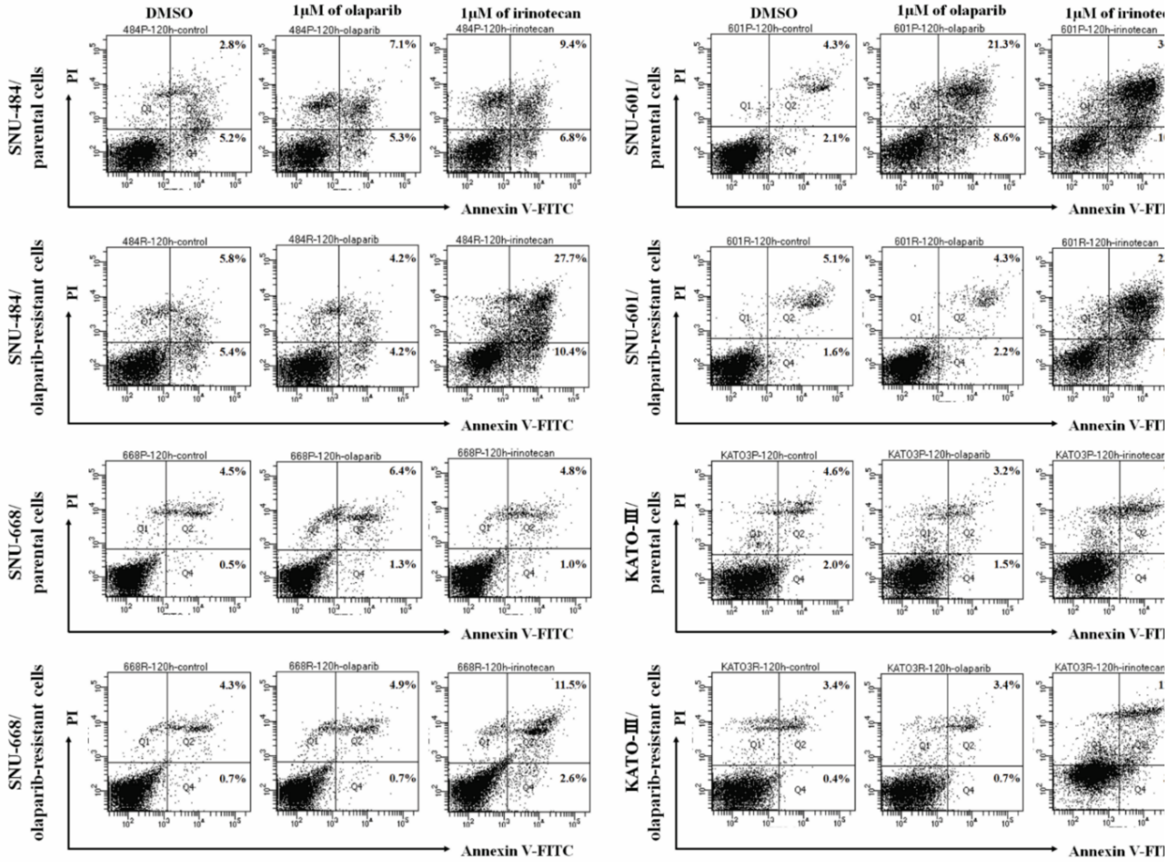

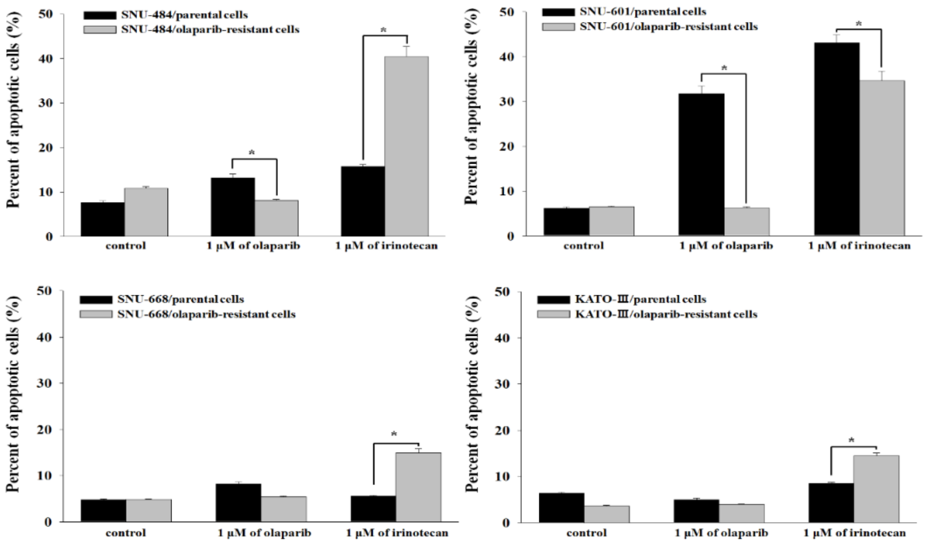

Figure S1. The populations of apoptotic cells following irinotecan or olaparib treatment. The percent of apoptotic cells were analyzed by performing Annexin V assay. The population of Annexin and PI dual positive cells were calculated and presented in a bar graph with SEs (n = 3), * $p < 0.005$.

Supplementary Figure S2

A

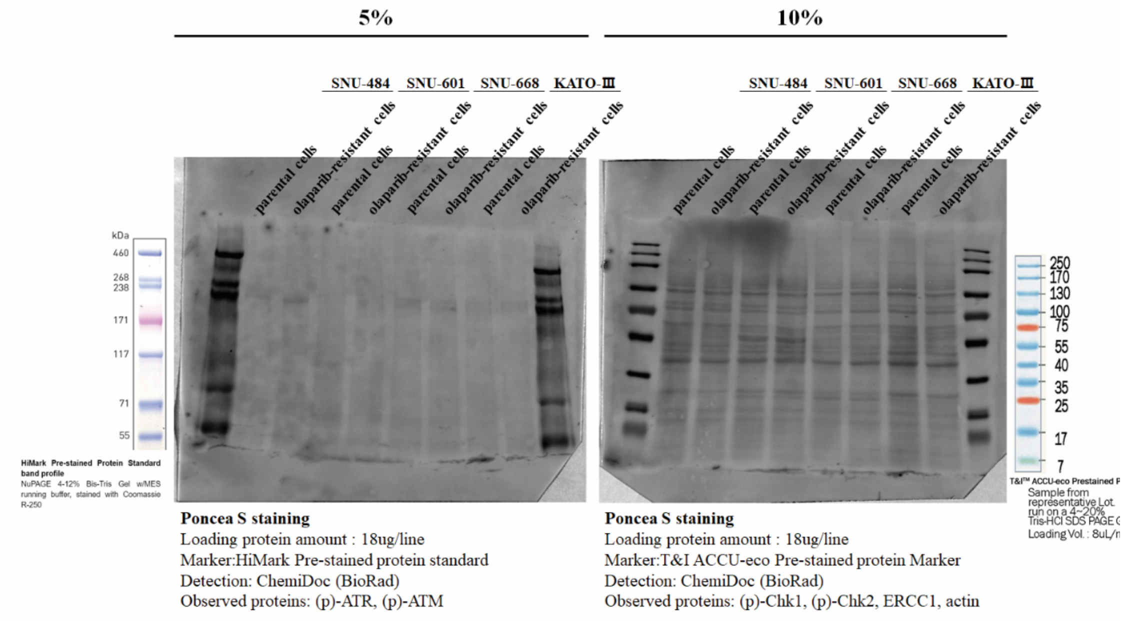

B

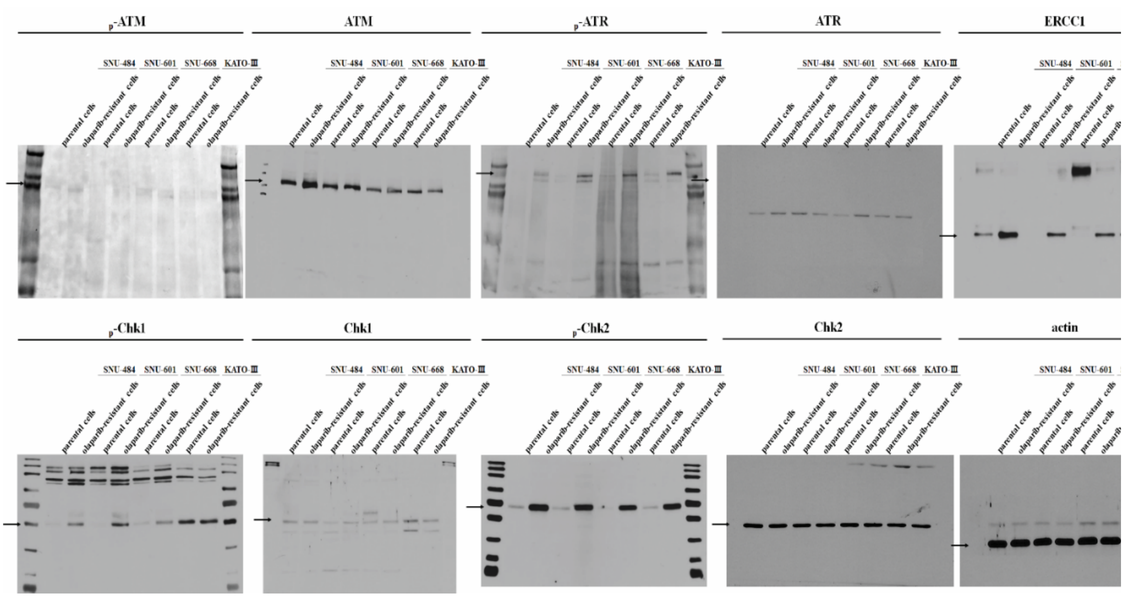

Figure S2. Protein expression levels of parental and olaparib-resistant cells. (**A**) Whole protein staining with Ponceau S reveals different expression levels of DDR molecules in parental and olaparib-resistant gastric cancer cells. (**B**) The basal expression levels of DDR proteins were detected in uncropped blots.

Supplementary Table S1. Sequences of the siRNAs.

gene	target sequence (5` → 3`)
non-specific	5′-AATTCTCCGAACGTGTCACG-3′
	5′-GGGAAGGACTCCATCAGATACTATA-3′
TOP1	5′-AAGTGGAAATGGTGGGAAGAA-3′
	5`-CGAAGAAGGTAGTAGAGTC-3`
	5`-GACCATATCTAGTAGTGAT-3`
TDP1	5`-GGAGTTAAGCCAAAGTATA-3`
	5`-CTAGACAGTTTCAAAGTG-3`
